# Supplementary material for: Multi-Omics Approach Reveals Redox Homeostasis Reprogramming in Early-Stage Clear Cell Renal Cell Carcinoma
Source: Antioxidants (Basel). 2022 Dec 29;12(1):81. doi: 10.3390/antiox12010081 (PMC9854847; doi:10.3390/antiox12010081)
Supplement: Supplementary file 1 [file antioxidants-12-00081-s001.zip › Supplementary Figures Legend.pdf]

Figure S1. Normalization effect of proteome and SNO-proteome. (**A**) Normalization effect on proteome data; (**B**) Normalization effect of proteome samples; (**C**) Normalization effect on SNO-proteome data; (**D**) Normalization effect of SNO-proteome samples.

Figure S2. Protein localization signatures of the proteome and SNO-Proteome. (**A**) Pie chart of SNO-proteins localization; (**B**) Pie chart of proteins localization.

Figure S3. Normalization effect of redox metabolites and samples. (**A**) Normalization effect of redox metabolites; (**B**) Normalization effect of samples.

Figure S4. SIMCA software validation of the REME data. (**A**) OPLS-DA analysis of the redox metabolites by SIMCA software; (**B**) OPLS-DA score plot by SIMCA software; (**C**) Permutation analytical validation of the OPLS-DA model; (**D**) VIP score plot of redox metabolites validated by SIMCA software; (**E**) S-plot of redox metabolites with SIMCA software.
