# Supplementary figures and images for: Multi-Omics Approach Reveals Redox Homeostasis Reprogramming in Early-Stage Clear Cell Renal Cell Carcinoma
Source: Antioxidants (Basel). 2022 Dec 29;12(1):81. doi: 10.3390/antiox12010081 (PMC9854847; doi:10.3390/antiox12010081)

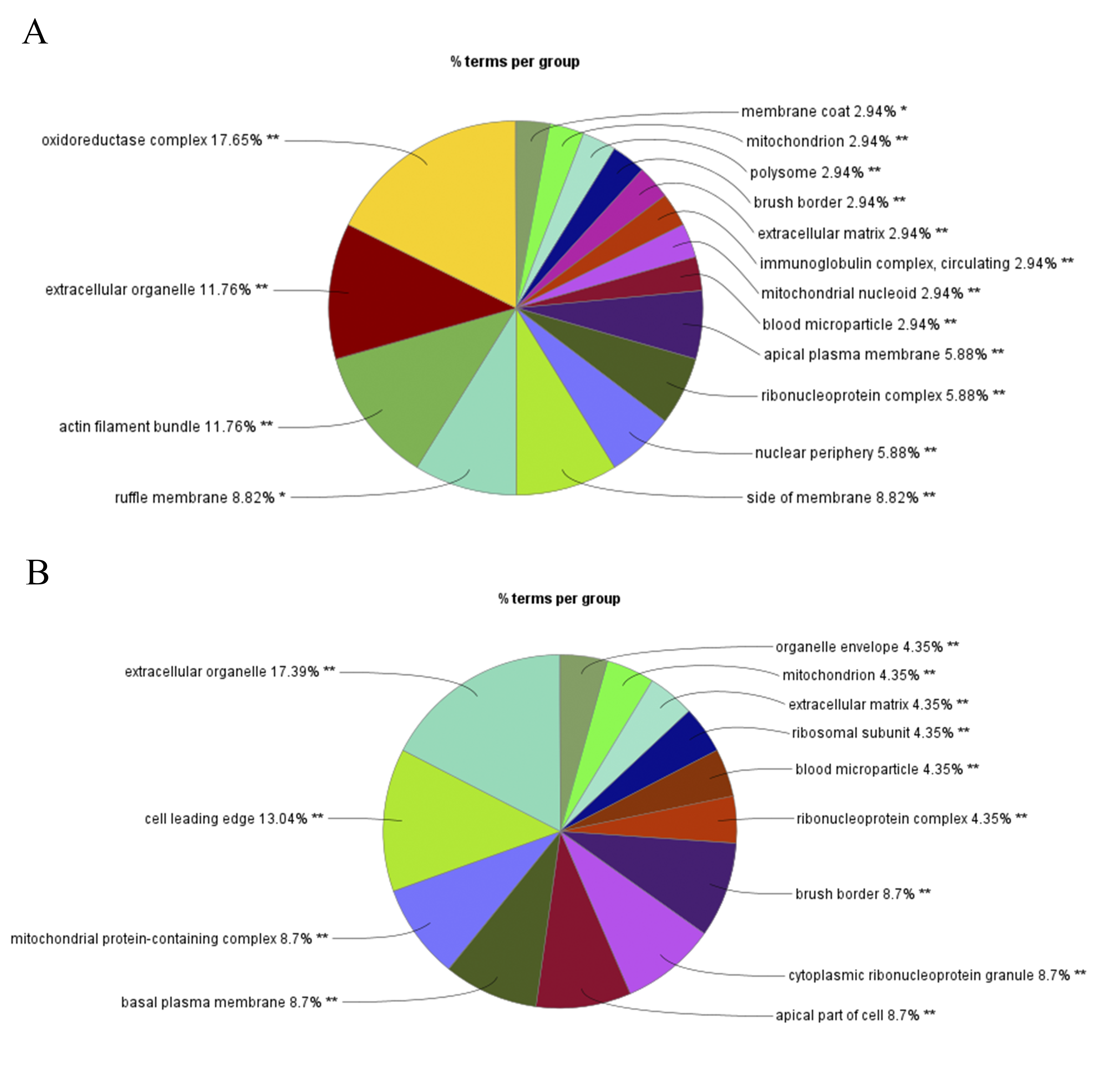

Supplement: Supplementary file 1 [file antioxidants-12-00081-s001.zip › Figure S2.tif]

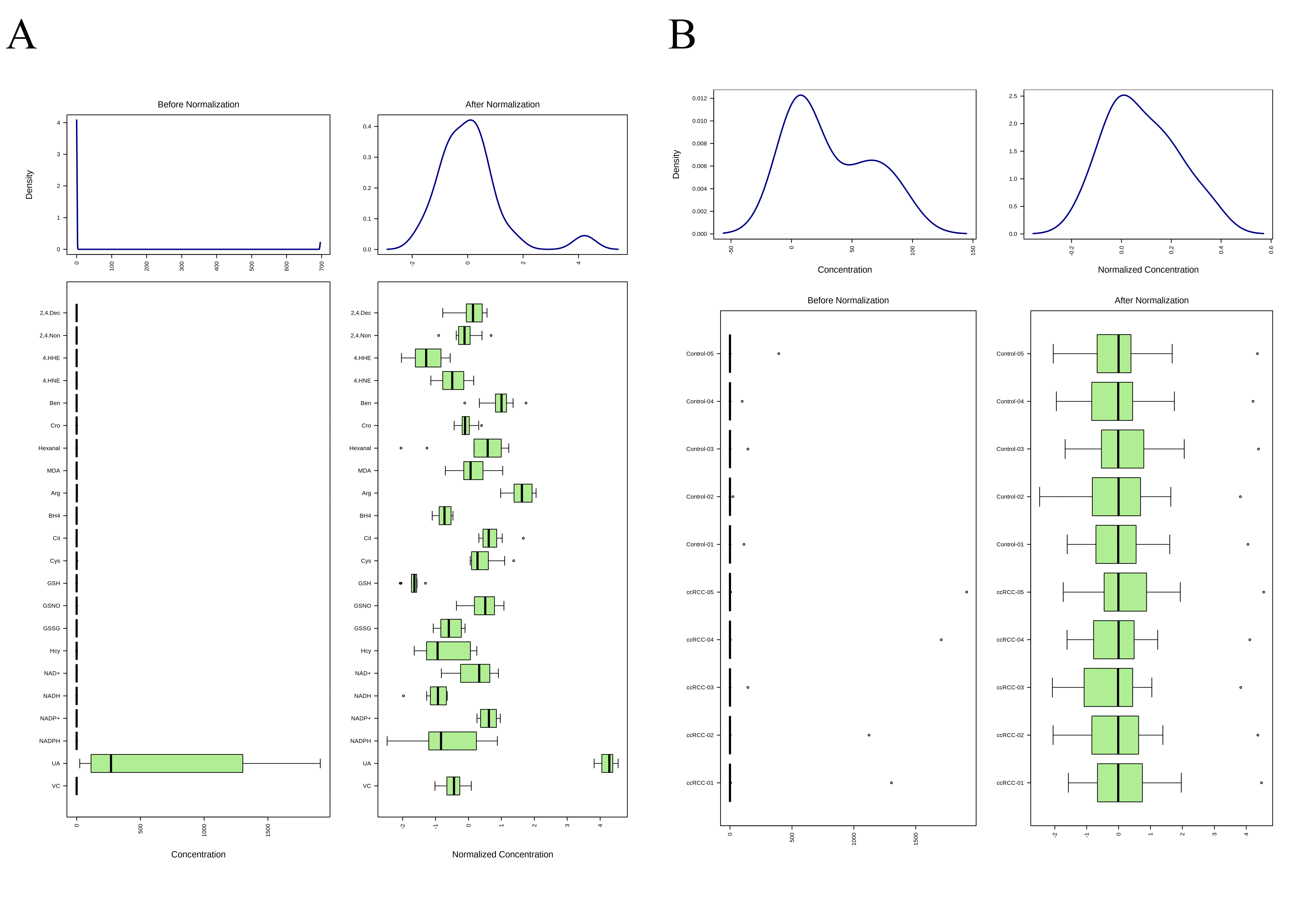

Supplement: Supplementary file 1 [file antioxidants-12-00081-s001.zip › Figure S3.tif]
